# Biomechanics of elite handcyclists are highly individual—a case series on kinetics, kinematics, and muscular activity

**DOI:** 10.3389/fspor.2025.1581301

**Published:** 2025-05-23

**Authors:** Oliver J. Quittmann, Carola Birk, Fabian Göll, Simon Nolte, Thomas Abel

**Affiliations:** ^1^ Department IV: Movement Rehabilitation, Neuromechanics and Paralympic Sport, Institute of Movement and Neurosciences, German Sport University Cologne, Cologne, Germany; ^2^ Institute of Biomechanics and Orthopaedics, German Sport University Cologne, Cologne, Germany

**Keywords:** surface electromyography (sEMG), fatigue, wheelchair athletes, cycling, VO2max, motion capture (Mocap), exercise testing, handcycling

## Abstract

**Aim:**

This study aimed to assess the biomechanics of handcycling propulsion under various exercise modalities in elite handcyclists with special emphasis on the work distribution between the push and pull phases.

**Methods:**

Three elite handcyclists in the H3/4 categories (women = 2) performed several lab tests on their own handbikes that were equipped with a powermeter (P9, SRM GmbH) to detect crank torque at 200 Hz. They performed a submaximal graded exercise test, a ramp test until exhaustion, two sprint tests, and a time-to-exhaustion trial at maximum aerobic power. Crank kinetics and joint kinematics were synchronized with surface electromyography of eight upper-extremity muscles.

**Results:**

The female athletes relied more on the pull phase, while the male handcyclist seemed to favor the push phase (∼10% more work). Shoulder and elbow flexion were almost unaffected by intensity, whereas other shoulder, wrist, and trunk angles changed individually. Even more differences were found in muscular activation patterns between athletes and they demonstrated high variability in the abdominals. During the time-to-exhaustion, we observed intensified work distributions (for the push and pull phases) and constant patterns. Muscular fatigue was identified in different muscles for the three athletes and covered the descending trapezius, abdominals, and anterior deltoid, respectively.

**Conclusion:**

These findings indicate that the biomechanics of handcycling propulsion are highly individual among elite handcyclists and may be influenced by their classification, handbike setup, and muscular capabilities. We encourage future research to replicate this study in a larger cohort and examine how work distributions and other biomechanical parameters change over time to individualize training prescriptions in athletes.

## 1 Introduction

Biomechanical research is essential in Paralympic Sports to gain profound insights into movements that are highly individualized. These insights allow for technical optimization, injury prevention, evidence-based classification, and improved performance (1). Measurements include assessment of kinetics, kinematics, muscular activity, and musculoskeletal modeling approaches. Even though research mainly focuses on technical optimization (1), combinations of biomechanical and physiological measures seem to be promising for holistic characteristics of human exercise (2).

Handcycling is a form of paracycling for athletes with lower-extremity impairments and/or spinal cord injury (SCI) who are unable to ride a conventional road bike or tricycle (3). After being included in the World Championships (1998) and Paralympic Games (2004), handcycling has gained popularity as a competitive and recreational sport (2, 3). Compared to manual wheelchair propulsion, it allows one to cover larger distances in a shorter period of time with less strain (4, 5).

Performance during time trials (15–22 km) in competitive handcyclists seems to be related to several physiological measures, including lactate threshold (*r* = 0.93), ventilatory thresholds (*r* = 0.92–0.96), peak oxygen uptake (*r* = 0.88–0.89), maximal aerobic power (*r* = 0.85), efficiency (*r* = 0.73–0.80), and relative bench pull (*r* = 0.77) and press (*r* = 0.70) (6, 7). It was found that persons with tetraplegia have a lower lactate threshold compared to persons with paraplegia (54 ± 15 vs. 111 ± 25 W), with similar differences observed between recreational (91 ± 21 W) and competitive (137 ± 15 W) handcyclists (8, 9).

Optimizing the handbike-user configuration was highlighted to be important in recreational and competitive users (2, 3). While ∼80% of recreational users found their pedal position to be optimal, ∼50% stated that their sitting position needed improvement (10). These statements highlight the need for an individualized handbike configuration among recreational users. In competitive handcyclists, comfort and stability were mentioned to be crucial considerations, whereas the support and padding solutions were found to be inadequate (11). A maximal crank distance of 97%–100% of arm length (acromion to the end of the fifth metacarpal) was found to be the most economical in terms of oxygen consumption (12), whereas a narrower distance of 94% was recommended to allow for a more even work distribution (13).

Synchronous handcycling propulsion is characterized by a cyclic transition between the push and pull phases. The whole cycle (360°) is usually characterized by the cranks pointing forward (0°), downward (90°), backward (180°), and upward (270°) (3). One revolution can be divided into six sectors that characterize the push (150°–330°) and pull phases (330°–150°) (14). The push phase includes the lift-up, push-up, and push-down sectors and the pull phase covers the press-down, pull-down, and pull-up sectors.

Most studies found a higher work distribution in the pull phase (13–18), whereas one study in competitive handcyclists observed a more productive push phase (19). Women (able-bodied) produced even more work in the pull phase compared to men (14). Changing the handgrip angle had a significant effect on the pull-down and lift-up contributions (14), whereas an increase in crank distance from 94% to 103% arm length resulted in an increase in pull phase distribution from 62% ± 7% to 69% ± 8% (13). With longer cranks, peak torque was higher and occurred earlier in the crank cycle (19). In able-bodied participants, the pull phase distribution increased during the course of a graded exercise test and during a 30 min constant load trial at lactate threshold (17, 20). In a recent review, inconsistencies in crank kinetics and joint kinematics between studies were traced to variability in reporting, experimental setup, handbike configuration, and the participants' experience (21).

Joint kinematics of handcycling seem to be affected by the gear ratio (22), crank position (13) and length (19), exercise intensity and duration (17, 20, 23), and performance level (9). Despite competitive handcyclists demonstrating more thorax flexion (∼5°), shoulder extension (∼10°), and posterior scapular tilt (∼15°), no differences in handbike configurations were found (9). In able-bodied participants, an increase in shoulder abduction and internal rotation and a decrease in shoulder and elbow flexion were negatively associated with performance in a graded exercise test (17). Cyclical motions of the limbs and joints are a consequence of corresponding muscular activity, which is predominantly assessed by surface electromyography (sEMG).

Muscular activity and its coordination profiles during handcycling propulsion have been studied in able-bodied novices (20, 23–25) and a single case of an elite handcyclist (26, 27). Faupin et al. (24) published the first holistic combination of synchronized crank kinetics, joint kinematics, and muscular activity at moderate intensity in an able-bodied participant. In a similar attempt, Litzenberger et al. (26, 27) assessed the effect of backrest and crank position on kinematics and muscular activity. While kinematics demonstrated only minor changes, a shift in muscular activity on/offset was found (26, 27). In the 10 years following this single case, there has not been a scientific update on elite recumbent handcycling.

This study aims to assess the biomechanics of handcycling propulsion under various exercise modalities in several elite handcyclists with a special emphasis on the work distribution between the push and pull phases. Thus, we build upon our extensive work in able-bodied participants under various exercise modalities (17, 20, 23, 25). Given the body of knowledge, it seems obvious that elite handcyclists have to be studied in their own individualized handbike, which makes measuring kinetics quite challenging. A measurement device that detects tangential torque at 200 Hz on the handbike seems to solve this problem. Based on our previous work, we hypothesize that the pull phase is used to a higher extent at (a) higher intensities and (b) during the course of muscular fatigue.

## 2 Methods

### 2.1 Participants

Three elite recumbent handcyclists (two women, one unilateral amputee, two athletes with an SCI) in the H3/4 categories participated in this study. All three athletes (P01, P02, and P03) were (former) medalists at the World Championships and/or Paralympics, including the 2024 Paris Games. P01 is a male H3 handcyclist with an SCI, P02 is a female H4 athlete with unilateral amputation, and P03 is a female H3 athlete with an SCI. All the tests were performed in 2023. The inclusion criteria were defined as recent participation in international events and/or membership of a national team. Medical peculiarities that prevented participants from exhaustive exercise and acute complaints of the upper extremities were the exclusion criteria. Athletes gave their written informed consent before participating in this study. The study was approved by Ethics Committee of the German Sport University Cologne (112/2023) and complied with the standards of the 1975 Helsinki Declaration modified in 1983.

### 2.2 Procedures

The tests were performed on their own recumbent racing handbike, which was mounted on a fully calibrated and validated ergometer (Cyclus 2, 8 Hz, RBM electronic automation GmbH, Leipzig, Germany). Crank arm length ranged from 155 (P02) to 165 mm (P01/P03). A powermeter (PM9, 200 Hz, Schobener Rad Messtechnik GmbH, Jülich, Germany) was installed on the handbikes to detect tangential crank kinetics and crank position at a high temporal resolution. A cooling fan was used during strenuous exercise testing.

The handcyclists performed all the tests on a single day with physiological exercise testing until noon and biomechanical measurements in the afternoon. After receiving a medical check-up, including a resting electrocardiogram, the participants performed a submaximal graded exercise test until lactate concentration exceeded 4 mmol/L. The test started with an intensity of 20 W and increased every 5 min by 20 W (17, 28). At the end of each step, 20 µl blood samples were taken from the right earlobe and analyzed using a stationary analyzer (Biosen C-Line, EKF-diagnostics GmbH, Barleben, Germany). The power output corresponding to a lactate concentration of 4 mmol/L was interpolated linearly (29). Throughout the graded exercise test, the participants wore an airtight silicone oro-nasal mask (7450 Series, V2™, Hans-Rudolph, Inc., Shawnee, KS, USA) to record oxygen uptake and carbon dioxide output breath-by-breath using a metabolic cart (ZAN 600 USB, nSpire Health, Inc., Longmont, CO, USA). After the graded exercise test, a 10 min cool-down at a self-selected intensity was performed.

After 1 h of rest, the participants performed a ramp protocol until voluntary exhaustion to determine maximal power output and maximal oxygen uptake. The ramp test started with an initial work rate of 80 W for 10 min, which increased by 5 W every 15 s. Post-exercise lactate concentration was recorded immediately after exhaustion, and at 3 and 6 min following passive recovery. Afterward, a 10 min cool-down at a self-selected intensity was performed, followed by a >2 h lunch break.

In the afternoon, the participants were prepared for motion capture and sEMG. A total of 50 retro-reflective markers were placed on the handbike, ergometer, and anatomical landmarks according to the UpperLimb-Model and these were tracked by infrared cameras (200 Hz, MF-F40/14, Vicon Motion Systems Ltd., Oxford, UK). Kinematics included shoulder flexion, abduction and internal rotation, elbow flexion, and wrist movements (palmar flexion and radial duction). In accordance with recommendations of the International Society of Biomechanics (ISB), trunk orientation in the sagittal plane was determined as previously described by using a marker array attached to the sternum (17).

Wireless sEMG electrodes (Delsys® Trigno™, Boston, MA, USA) were used to record muscular activity of the M. biceps brachii (BB); M. triceps brachii (TB); M. deltoideus, Pars clavicularis (DA); M. deltoideus, Pars spinalis (DP); M. pectoralis major, Pars sternalis (PM); M. trapezius, Pars descendens (TD); M. rectus abdominis (RA); and forearm flexors (FC). Electrode positions and skin preparation were in accordance with the guidelines of surface electromyography for the non-invasive assessment of muscles (SENIAM) (30). Muscular activity patterns were assessed as the crank angles where the sEMG values exceeded a threshold of 30% of a muscle's local amplitude/range (max–min) (26, 27). The on- and offsets of this threshold and the range of activation demonstrated good to excellent reliability if 6–10 consecutive revolutions were averaged, respectively (25).

The normalization of sEMG signals was in accordance with previous research, indicating that sport-specific maximum voluntary isometric contractions (MVICs) are suitable for the TB, DA, and DP (31). During these tasks, the ergometer was blocked with a steel pin that was placed in a hole in the ergometer's brake disk (31). Accordingly, the participants performed three MVICs for 2 s each at fixed crank angles of 0°, 90°, 180°, and 270°. Simultaneously, crank torque measurements were recorded as sport-specific strength testing. Furthermore, muscle-specific normalizations were performed for the FC (hydraulic hand grip dynamometer), TD (shoulder elevation task), BB (elbow flexion task), and RA (trunk flexion task) (31).

For biomechanical measurements, the participants performed two 15-s all-out sprints that started at 0° (first sprint) and 180° (second sprint), respectively. This was due to the fact that (1) handcyclists tend to use different starting positions in a race and (2) the starting position may influence kinetics, kinematics, and muscular activity. Between the sprints, the athletes recovered for >10 min. Afterward, the participants performed three trials each at low and medium intensity at 50% and 100% of lactate threshold power, respectively. Finally, a high-intensity and fatiguing time to exhaustion test was performed at the maximal power attained in the ramp test. Work distributions were averaged in quartiles (Q1–Q4) to compare the athletes during this trial. In other words, we quantified the average work distribution from 0% to 25%, 25% to 50%, 50% to 75%, and 75% to 100% of the individual time to exhaustion to account for potential fluctuations in work distribution. To provide a more practical illustration in a bar chart, work distribution was expressed as a percentage that tended toward the push (positive values) and pull phases (negative values). For example, if 60% of the rotational work is performed in the push phase, a value of +10% would be illustrated. Conversely, if 55% of the rotational work is performed in the pull phase, a value of −5% would be illustrated.

### 2.3 Processing

Data processing was performed in RStudio 4.1 and augmented by several packages and Python scripts that are available in the provided code. Spirometric data were averaged for the last 30 s of each step of the graded exercise test. The fractional utilization of oxygen uptake at 4 mmol/L lactate concentration was linearly interpolated. For the ramp test protocol, a fourth-order, zero-lag Butterworth filter was applied to determine maximal oxygen uptake (32). In terms of the power data, the first (in-) complete revolution and the last two revolutions of each submaximal trial were not used for data extraction. The only exception to this was the sprint tests, which included the first revolution.

Biomechanical analog data were converted from .c3d to .csv using Python code. sEMG data were rectified and filtered with a 200-ms moving average (31). Crank kinetics were synchronized to the motion capture and sEMG by detecting the initial movement of the cranks (which was recorded in both systems). This was possible as the sEMG units have three-axis accelerometers installed. As such, biomechanical measurements had to start from a stationary position and were recorded for approximately 5 s before the actual (∼25 s) trial started. Synchronization was applied using the first optical reference of movement in the motion capture (marker on the cranks) and sEMG unit acceleration (forearm). All the trials, especially the MVICs, were visually inspected by two independent examiners to ensure usefulness. The sEMG data were rectified, smoothed using a 200-ms moving average, and normalized to the highest amplitude recorded in any of the MVIC trials for each muscle. Kinematics were processed according to previous work by using a fourth-order low-pass Butterworth filter with a cut-off frequency of 0.2. Given the fairly low sample size, we did not perform inferential statistics and focused on a visual representation/interpretation of elite handcyclists' biomechanics. The datasets, codes, and analyses for this study can be found on GitHub.

## 3 Results

The handcyclists demonstrated a lactate threshold of 140 ± 33 W, a maximal oxygen uptake of 46.6 ± 6.9 ml/min/kg, and a fractional utilization of 69 ± 18%. Time to exhaustion at maximum ramp test power was ∼1 min for all athletes.

### 3.1 Work distribution across trials and sectors

**Figure 1:**
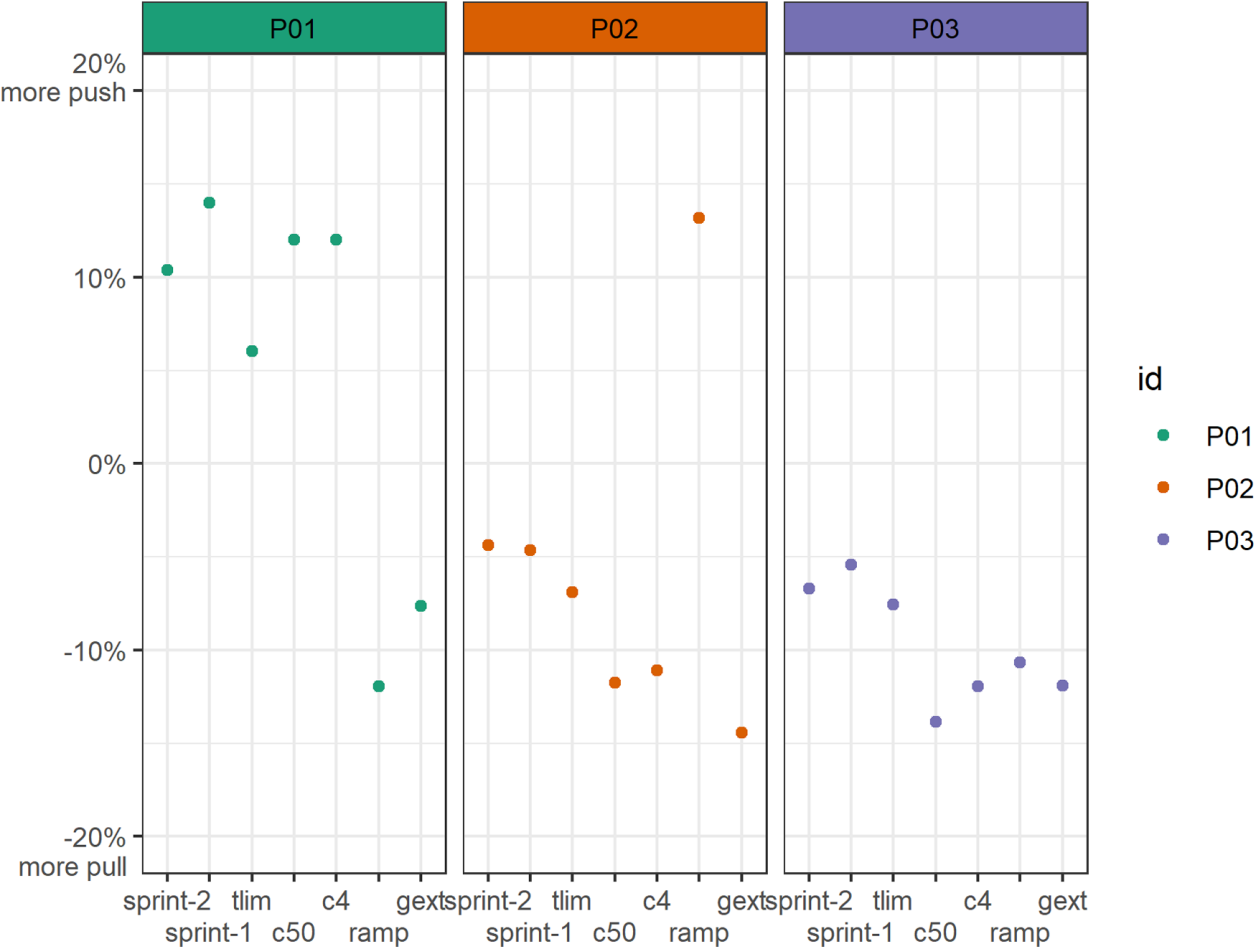
Work distribution profiles of all participants and conditions in terms of the push and pull phases. The push phase was defined as crank angles of 150°–330°. A value of +10% represents a dominant push phase of 60%, whereas a value of −15% represents a dominant pull phase of 65%. c4, power output according to a blood lactate concentration of 4 mmol/L; c50, power output equivalent to 50% of c4; gxet, graded exercise test (average); ramp, ramp test (average); sprint-1, first sprint starting at a crank angle of ∼0° (initial pull); sprint-2, second sprint starting at a crank angle of ∼180° (initial push); tlim, time to exhaustion test (average).

Except for the ramp test in P02, P02 and P03 demonstrated a more pronounced pull phase with values ranging from −5% to −15% (Figure 1). In contrast, P01 tended to produce more work during the push phase (∼10%) in the afternoon trials, whereas the opposite was observed in the average distribution during the graded exercise and ramp test. Differences between sprints (0° and 180°) were fairly low, except for P01, who demonstrated a 4% reduction of push-phase distribution when starting in the 180° (initial push) position.

**Figure 2:**
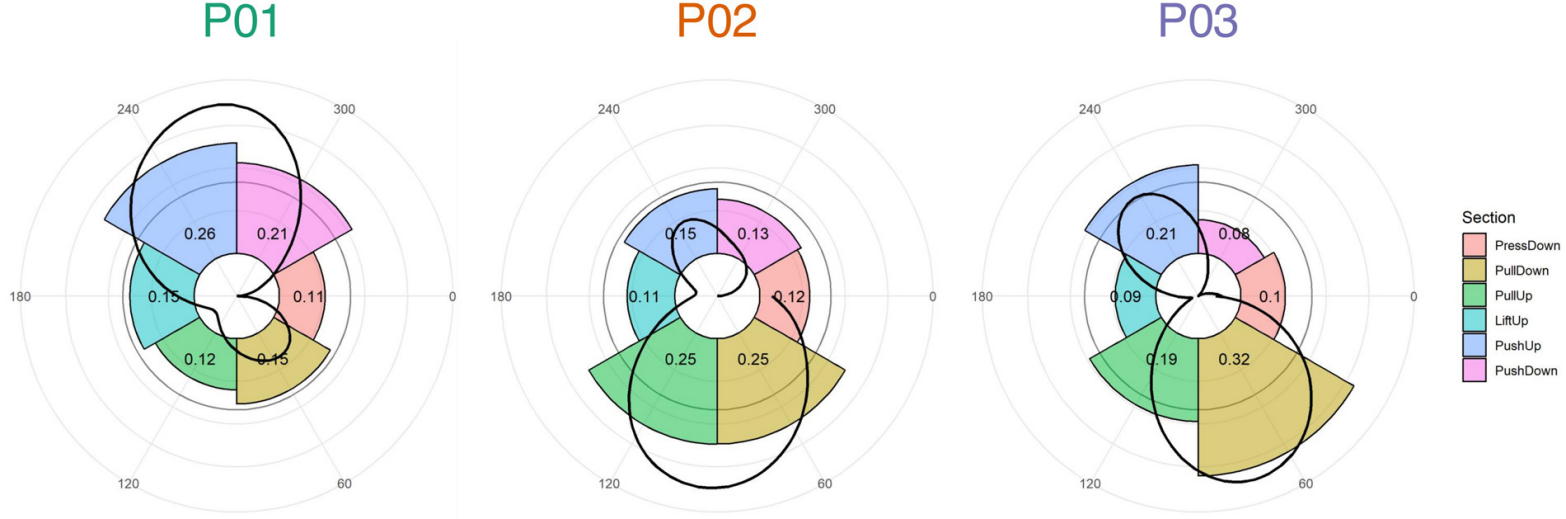
Mean torque profiles (black line) and section distributions (colored bars) at the onset of blood lactate accumulation (4 mmol/L). The bars illustrate the relative distributions of the six sections in the whole rotational work distribution. Their relative contribution is also displayed as numeric decimals. A value of 0.20, for example, indicates that this section distributes a total of 20% of the total rotational work, which is higher than an even distribution of 1/6 = 16.66%. Sections were determined according to previous studies (14, 17).

Work distributions across the six sectors demonstrated substantially more variability between the handcyclists (Figure 2). Figure 2 illustrates the average torque and sector distributions for all the participants at their individual lactate threshold (4 mmol/L). The average push-to-pull ratios of P01 (62/38), P02 (39/61), and P03 (38/62) indicated similarities in the female handcyclists. Even though the average pull sector distribution was approximately 20% in P02/03, the sector distributions differed by up to 7%. P02 had a more even distribution in the pull-down and pull-up sectors (25% each), whereas P03 had a higher pull-down (32%) and lower pull-up (19%) contribution. Similarly, P03 had higher push-up (21%) and lower push-down (8%) contributions compared to P02 (15% and 13%, respectively). Conversely, P01 had even higher push-up and push-down contributions of 26% and 21%, respectively. However, all the handcyclists demonstrated fairly low press-down (10%–12%) and lift-up (9%–15%) contributions.

### 3.2 Kinematics and muscular activity across intensities

**Figure 3:**
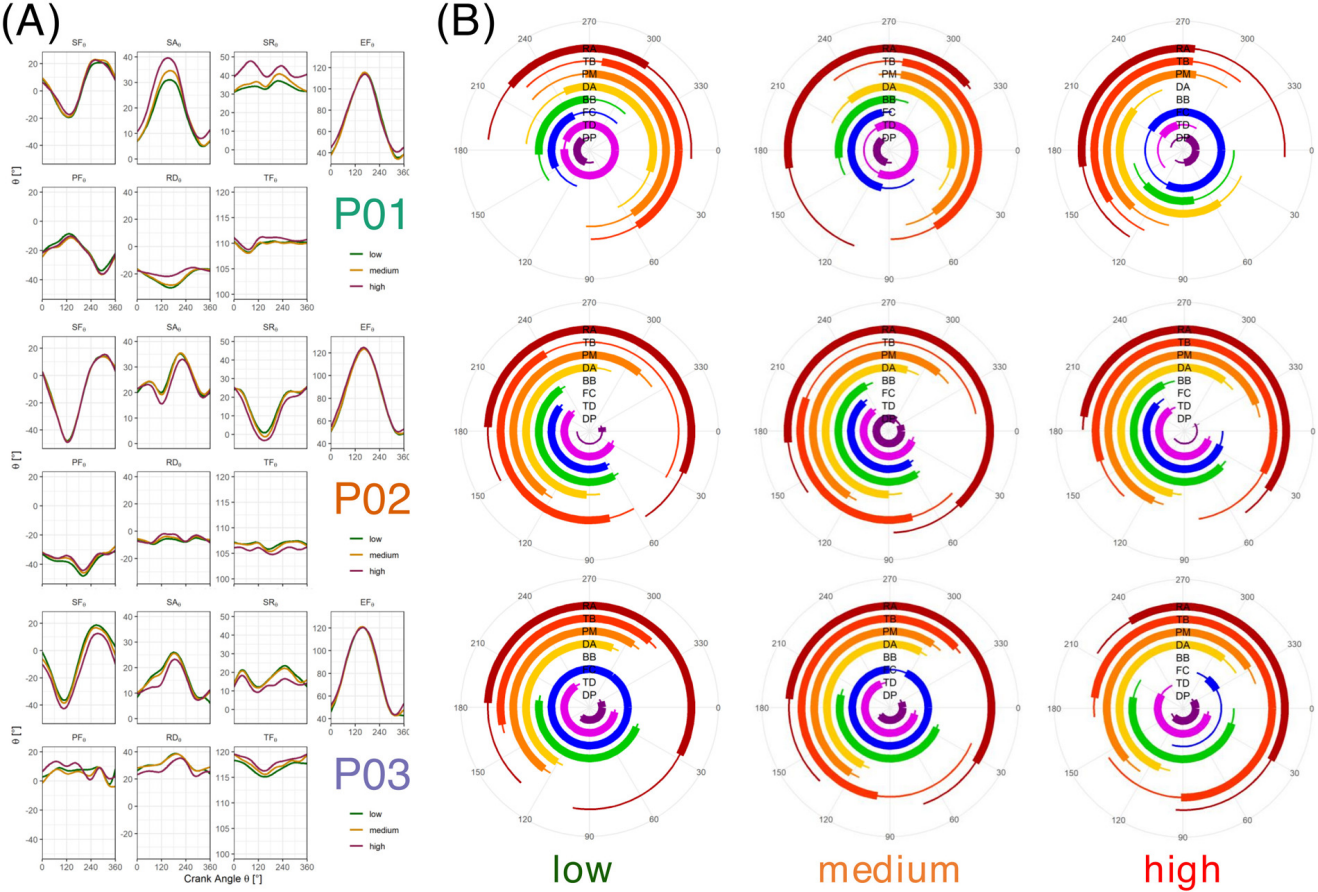
Joint kinematics **(A)** and muscular activation patterns **(B)** at low, medium, and high intensity. Low intensity was defined as 50% of the power output corresponding to a 4 mmol/L lactate concentration. Medium intensity was identified as the onset of the blood lactate concentration (4 mmol/L). High intensity was identified as the maximum power output attained in the ramp test protocol. BB, M. biceps brachii (Caput breve); DA, M. deltoideus (Pars clavicularis); DP, M. deltoideus (Pars spinalis); EF, elbow flexion; FC, M. flexor carpi radialis (forearm flexors); PF, palmar flexion; PM, M. pectoralis major (Pars sternalis); RA, M. rectus abdominis; RD, radial duction; SA, shoulder abduction; SF, shoulder flexion; SR, shoulder internal rotation; TB, M. triceps brachii (Caput laterale); TD, M. trapezius (Pars descendens); TF, trunk flexion.

Joint kinematics demonstrated similar results across handcyclists for elbow and shoulder flexion, which were hardly affected by exercise intensity (Figure 3A). In contrast, intensity seemed to affect shoulder internal rotation, shoulder abduction, trunk flexion, and wrist movement the most. Interestingly, shoulder internal rotation and abduction largely increased in P01 and decreased for P02/03 with increasing intensity. Based on trunk angle, P02 had a more upright position.

Inter-individual variability was found to be even higher with respect to muscular activity profiles (Figure 3B). Despite an earlier onset of the BB and TD in P02 and an earlier onset of the DA and TB in P03, these athletes tended to show comparable activation profiles. In contract, P01 demonstrated completely different profiles—especially at low and medium intensity. With increasing intensity, P02 had an earlier onset of the TD, FC, BB, and PM, whereas P03 had a later onset of the PM and a shorter period of co-contraction between the BB and TB.

### 3.3 Work distribution and muscular activity during the time to exhaustion trial

**Figure 4:**
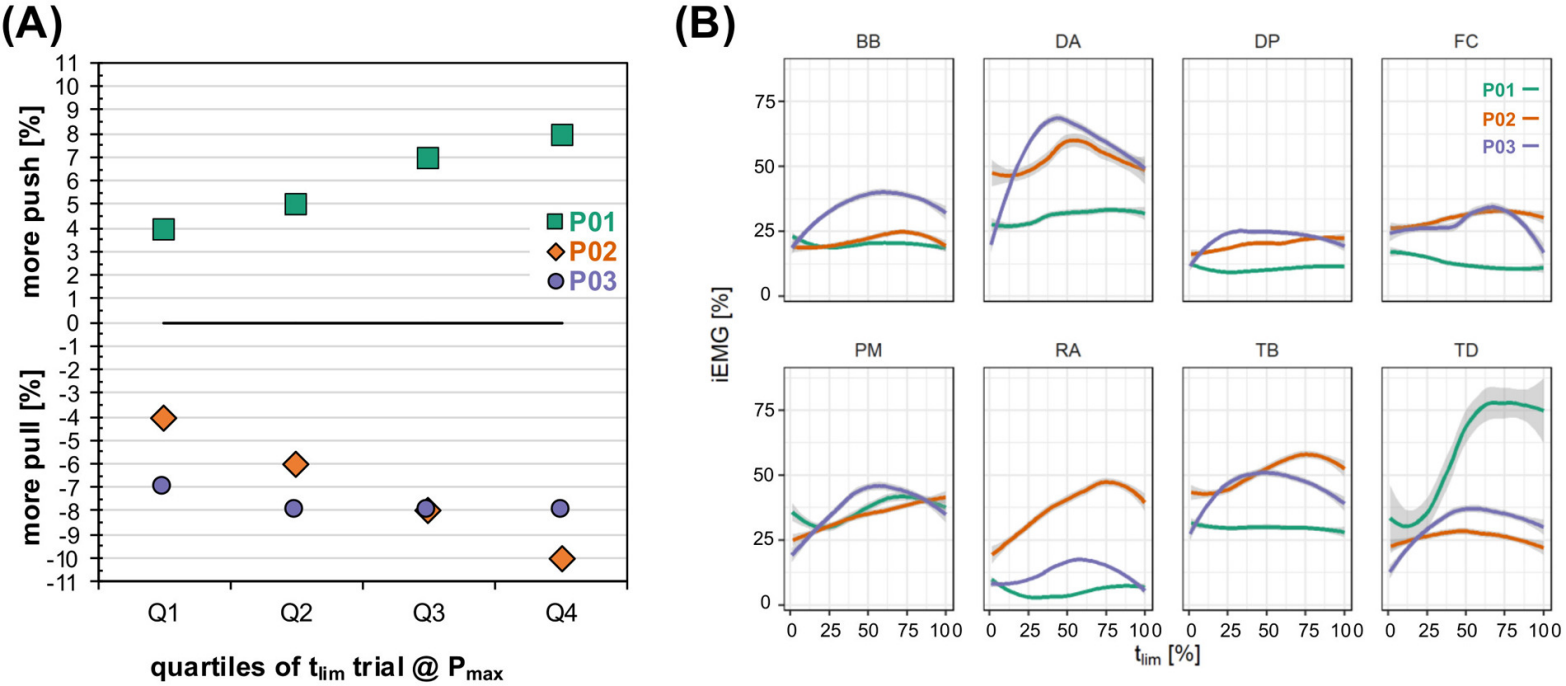
Work distributions **(A)** and muscular activity **(B)** during the course of the time to exhaustion trial. The intensity was individualized as the highest power output attained in the ramp test protocol. BB, M. biceps brachii (Caput breve); DA, M. deltoideus (Pars clavicularis); DP, M. deltoideus (Pars spinalis); FC, M. flexor carpi radialis (forearm flexors); PM, M. pectoralis major (Pars sternalis); Q 1, first quartile; Q2, second quartile; Q3, third quartile; Q4, last quartile; RA, M. rectus abdominis; TB, M. triceps brachii (Caput laterale); TD, M. trapezius (Pars descendens); t_lim_, time to exhaustion.

Work distributions demonstrated a more pronounced push phase (P01), a more pronounced pull phase (P02), and a constant distribution (P03) during the course of the ∼1 min time to exhaustion test (Figure 4A). Whereas P01 shifted from +4% to +8% more push, P02 shifted from −4% to −10% more pull. In contrast, P03 kept the distribution at −7% to −8% more pull.

Muscular activity demonstrated rather heterogeneous activation behavior between the participants (Figure 4B). P01 demonstrated a substantial increase in TD activity (up to >75% MVIC), whereas P02 had a huge increase in RA activity (almost 50% MVIC). Interestingly, P03 demonstrated a reverse U-shape activation that peaked at approximately 50% of the time to exhaustion for the DA, BB, TB, PM, TD, and RA. While power output was maintained during the trial of P03, the cadence substantially dropped at this point.

## 4 Discussion

To the best of our knowledge, this is the first study to report the kinetics, kinematics, and muscular activity during handcycling propulsion under various exercise modalities in several elite handcyclists. We found that the work distribution varied between athletes, intensities, and fatigue states. The differences were even more pronounced when illustrating the sector distributions. Exercise intensity affected the joint angles outside the sagittal plane the most, but these alterations were not consistent between the athletes. The same was true for muscular activity profiles that demonstrated an earlier onset of some muscles at higher intensities. During a short-term (high-intensity) fatiguing time-to-exhaustion trial, all *n* = 3 handcyclists coped differently and demonstrated increased muscular activity on an individual level. This indicates that peripheral muscular fatigue is highly individual, even in elite handcyclists.

### 4.1 Work distribution

In accordance with previous research in able-bodied participants (14), the female handcyclists produced substantially more work during the pull phase. Even though this may be related to lower arm extensor strength in women, this specific group of participants does not allow for a generalized explanation. The fact that one of the athletes was classified as an amputee handcyclist may be responsible for the higher reliance on the pull phase. It has been found that athletes with normal lower limb function who are able to perform a closed-chain (the feet are actively pressing against the footrests) had a +11% higher pull performance, when compared to those without lower limb function (33). However, this does not apply to the other female athlete, who had a similar high reliance on pull phase contribution.

The higher work distribution in the push phase seems to fit with previous findings in competitive male handcyclists (19). We did not observe a higher reliance on the pull phase in the athletes with a higher crank force-aft position, as experimentally highlighted in able-bodied athletes (13). In contrast to previous findings in able-bodied males (17, 20), the elite handcyclists did not demonstrate an increased pull phase with higher exercise intensity and duration. This could indicate that years of experience lead to highly individual coping mechanisms that are not present in able-bodied novices. Given the fairly easy measurement of crank kinetics, these findings can be applied to real-life recordings during races.

### 4.2 Joint kinematics

In accordance with previous studies that demonstrated differences in kinematics between recreational and competitive handcyclists (9), the results of this study seem to (slightly) differ from those in able-bodied participants (17). An increase in shoulder abduction and internal rotation and a decrease in elbow and shoulder flexion with increasing power output were only observed in one of the participants (17). Hence, the results of the previous work do not seem to apply to (all) elite handcyclists. The position of the highest and lowest elbow flexion was shifted compared to studies in able-bodied participants (17, 20, 23) and more aligned with findings in an elite handcyclist (26, 27). This is most likely due to the even more recumbent position of the handcyclist on current racing handbikes. Given the fact that elbow and shoulder flexion and shoulder abduction are affected by crank length (19), the data of this study were probably affected by the individualized handbike setups, which hinders a standardized comparison between the athletes. The measurement of joint kinematics in handcycling relies mostly on an extensive setup in laboratories and is thus less accessible when compared to crank kinetics and sEMG.

### 4.3 Muscular activity

Muscular activation profiles demonstrated the highest inter- and intra-subject variability among all the measurements. On the one hand, this may be due to the fact that sEMG is highly sensitive to sweat contamination and thus prone to artificial signals. On the other hand, it may be due to highly individualized participant characteristics. It is difficult to compare these findings to the single elite handcyclist case (26, 27) and to able-bodied novices (20, 23, 25). Given that activation profiles shift with different crank positions (27), this aspect definitely hinders a comparison. However, especially for the BB and PM, our results seem to align with previous studies (25, 27), except for the first published able-bodied single case (24).

In contrast to previous work in able-bodied participants (20, 23, 25), a substantially increased and altered activation profile of the DP with exercise intensity and duration was not observed in the elite handcyclists. This is an interesting finding as it indicates that this rather small muscle, which initiates the pull phase, may not be a deficit in highly trained handcyclists when compared to recreational able-bodied triathletes. We know that all the included participants in the present study performed regular resistance exercise in their training. We speculate that the yearly exposure to handcycling and accompanying resistance training diminishes the deficits of smaller muscle groups compared to large muscle groups. Hence, combining measures of crank kinetics (including crank position) with sEMG allows us (a) to understand the interplay between muscles during different phases of propulsion and (b) to assess how activity profiles are affected by exercise intensity, duration, and fatigue. We argue that this may explain most of the individuality in propulsion characteristics, which may be improved with certain training protocols.

### 4.4 Limitations and future directions

Given the low sample size of *n* = 3 athletes and the consequently explorative nature of this report, these results cannot be generalized to all elite handcyclists. However, these findings indicate that even on a highly experienced level, biomechanical measures and coping strategies can substantially vary across practitioners. Another limitation arises from the one-dimensional crank kinetics. Having the opportunity to record 3D-kinetics on the handcyclists own handbike would allow for further insights such as symmetries and fractional effective force (15, 16). In this study, we evaluated a time-to-exhaustion of approximately 1 min. Even though this seems to be useful for examining fatigue, this is clearly below the demands of team relay events that typically cover several high-intensity bouts of approximately 3 min. Hence, expanding the spectrum of trials is necessary to gain even more specific insights. Another expansion worth exploring is musculoskeletal modeling that can be fitted with data similar to that in this study (21). In addition, combining biomechanical and physiological measurements allows for more insights in the mechanics and energetics of handcycling propulsion (1, 2, 12).

As with all Paralympic sports, individuality and heterogeneity across athletes (even within the same category) make standardized comparisons challenging, especially in a field that requires a mechanical setup. However, we did not control for crank position in terms of arm length or handbike model. Another aspect worth investigating is the comparison of left and right sides in terms of kinematics and muscle activity, as already performed in a single case (26, 27). Coupled with unilateral strength testing, this would allow to evaluate if and why certain asymmetries exist. Initially, we hoped to measure athletes twice to assess modifications over time. Thus, we encourage future studies to examine how these biomechanical aspects change due to training. Recently, modeling kinematics during handcycling using inertial measurement units and a temporal convolution network was found to result in reasonable results that may be more easily applied in real-world settings (34). At least in terms of crank kinetics, the implemented powermeter allows for field-testing, even during major events, and, as such, may guide coaches and athletes to improve performance.

In conclusion, this case series indicates that the biomechanical aspects of handcycling propulsion are highly individual among elite handcyclists. Differences between athletes were observed in kinetics, kinematics, and, most notably, muscular activity. It seems that different mechanisms of muscular recruitment and/or fatigue underlie the patterns observed in exhaustive exercise. These findings could be helpful to improve individualized conditioning and training among handcyclists. In particular, measures of crank kinetics can be recorded outside the laboratory and provide real-life data on the road that are of particular interest for improving individual technique, strategy, and pacing. However, given the small sample size and heterogeneity in terms of handbike configuration, classification, and sex, we cannot draw generalized conclusions.

## Data Availability

The datasets presented in this study can be found in online repositories. The names of the repository/repositories and accession number(s) can be found in the article/Supplementary Material.
